# Clustering of diet, physical activity and sedentary behavior among Brazilian adolescents in the national school - based health survey (PeNSE 2015)

**DOI:** 10.1186/s12889-018-6203-1

**Published:** 2018-11-21

**Authors:** Thiago Sousa Matias, Kelly Samara Silva, Jaqueline Aragoni da Silva, Gabrielli Thais de Mello, Jo Salmon

**Affiliations:** 1 Department of Physical Education, School of Sports, Federal University of Santa Catarina, Florianópolis, Brazil; 2 School of Exercise and Nutrition Sciences, Deakin University, Melbourne, Australia

**Keywords:** Cluster analysis, Diet, Exercise, Sedentary lifestyle, Adolescent

## Abstract

**Background:**

There is a lack of evidence regarding clusters of health-related behaviors among adolescents from low, lower-middle, and upper-middle income countries. This study aimed to identify clustering patterns of health-related behaviors (diet, physical activity [PA] and sedentary behavior [SB]) and association with sociodemographic variables among a population-based sample of Brazilian adolescents.

**Methods:**

Cross-sectional data from the 2015 National School-Based Health Survey (PeNSE). A total of 102,072 (females: 51.7%) students in ninth-grade (age: 14.3 ± 1.1 years-old) enrolled in public and private schools were investigated in this study. Healthy and unhealthy diet, PA and SB were measured using a validated questionnaire. Two-step cluster analysis was conducted to identify lifestyle patterns. The methodology for complex analysis and weighting was used to inferential statistical procedures. Multinomial logistic regression assessed associations between sociodemographic factors and the clusters.

**Results:**

Three reliable and meaningful clusters were identified and labelled as follows: (1) health-promoting SB and diet (32.6%); (2) health-promoting PA and diet (44.9%), and (3) health-risk (22.5%). Compared to boys, girls were less likely to be in clusters 1 (OR = 0.85; 95% CI = 0.78–0.93, *p* < 0.001) and 2 (OR = 0.43; 95% CI = 0.40–0.46, *p* < 0.001) than the health-risk cluster. Higher socioeconomic status was positively associated with health-promoting PA and diet, and negatively related to health-promoting SB and diet. Older adolescents were more likely to be in cluster 1 than in cluster 3, compared to younger adolescents.

**Conclusion:**

Approximately one-quarter of the population (health-risk cluster) reported engaging in multiple risk behaviors. Interventions may need to be tailored to specific adolescent groups, especially considering sociodemographic differences.

**Electronic supplementary material:**

The online version of this article (10.1186/s12889-018-6203-1) contains supplementary material, which is available to authorized users.

## Background

Low levels of physical activity (PA), high levels of sedentary behavior (SB) and poor dietary habits are important contributors to several adolescent health problems, such as obesity and cardiovascular risk factors [1]. However, these behaviors do not occur in isolation and there is often synergy between them [2, 3]. An important issue that needs more attention is to investigate the combined occurrence of these behaviors [4, 5]. Indeed, a combination of behaviors will be able to better predict an individual’s overall healthy lifestyle [6–8].

Research on clustering of health behaviors has increased in recent times [9, 10] since it helps to deepen the understanding of how to promote health, allowing a more integrated approach [11]. Cluster analysis is a potential tool for organizing individuals into mutually exclusive groups by taking into account similarities in characteristics and behaviors [5, 9]. In this way, it is possible to verify which behaviors coexist among individuals [4].

A previous review showed that diet, PA, and SB tend to cluster in adolescents in a complex way, resulting in both healthy and unhealthy groups [5]. Also, observational studies have explored clustering patterns and thus observed a co-occurrence of positive and negative health-related behavior [6, 12–14]. For instance, in a study from 10 European countries of 2084 adolescents, the authors identified mixed clusters of unhealthy and healthy behavioral patterns (e.g. The cluster 3 was observed to be comprised by active adolescents, however, with low diet quality and high sedentary level; contrarily, cluster 4 was composed by inactive adolescents, however, with high diet quality and low sedentary levels) [4]. A study with 7372 children aged 9–11 years put out a concern due to a notable commonality in health-related behavior patterns was found across the 12 countries: all of them presented a cluster characterized by high levels of SB [15].

Despite this evidence, a recent systematic review called attention to the fact that most research has involved the examination of PA and SB, but not dietary factors [5]. That review reported 18 studies involving these behaviors, but only two included PA and diet and none included SB and diet. In addition, most research on clustering of these health behaviors comes from high income countries (e.g. USA, UK, Australia, and Canada). As the cluster patterns may be unique to particular cultures [8, 12], there is a need to investigate this in low income (e.g. Kenya), lower-middle income (e.g. India), and upper-middle income countries (e.g. Brazil, China, and Colombia).

There is also a lack of evidence regarding whether clusters of these health-related behaviors are only present in some sociodemographic groups [5, 8, 9, 16], or whether clusters of unhealthy behaviors appear to be more likely to occur among some sociodemographic groups and not others [11]. Previous findings showed that cluster differed by sex [6, 8, 9, 11, 17, 18], age [8, 11, 18], social class [11] and maternal education levels [8, 17]. The identification of which adolescents are more likely to engage in unhealthy behavior patterns allows us to understand which subgroups may be most at risk in terms of short- and long-term health outcomes [9]. The discrimination of population when associated to sociodemographic indicators, may help to better recognize and appropriately select strategies for obesity prevention [5].

The purpose of this study was to identify clustering of diet, PA and SB and association with sociodemographic variables among a national population-based sample of Brazilian adolescents. Such information might guide the development of health promotion programs, allowing a more targeted approach.

## Methods

### Study design and participants

Data from the third wave of the “National School-based Health Survey (PeNSE)” conducted in 2015 was used in this study. PeNSE used a cross-sectional design and was developed based on World Health Organization (WHO) recommendations for student health survey and is part of the Brazilian Surveillance of Risk and Protection Factors for Chronic Diseases. The main objective of the survey is to follow aspects of health and lifestyle behaviors of adolescents in public and private schools in Brazil.

Since 2009, the survey has been conducted every three years by the Ministry of Health and the Institute of Geographic and Statistics (IBGE) with support by the Ministry of Education (MEC). In first two waves (2009 and 2012) only students in ninth-grade (in Brazil, Elementary School includes ninth-grade, usually age ranges from 13 to 14 years old and is equivalent to freshmen in United States High Schools) participated. However, in Brazil there are a notable age-grade distortion and age ranged from 11 to 19 years old (see Additional file 1). In 2015, two age groups were investigated; ninth-grade and high school students (High School students in Brazil is equivalent to grades 10 through 12 in United States). In the present study, only students of the ninth-grade will be examined. This research was approved in National Committee of Ethics in Research: number 1.006.467/2015. Participation of all subjects was in accordance with ethical guidelines.

The sample was represented by the five geographical areas and also by the 26 capitals and the federal district of Brazil, representing the country as a whole. The sampling framework used the 2013 School Census database, and the sampling strategy included geographical stratification and multi-stage selection. The total geographical stratification was 53 (26 out of the capitals and 27 at the capitals). In all state capitals and the Federal District, the primary and secondary sampling units were schools and classrooms, respectively. School selection was proportional to the total number of ninth-year classes, while the classes in each school were chosen by simple random selection (schools with: < 2 9th grade = one classroom selected; ≥2 9th grade = two classrooms selected). The sizes of the samples were calculated to provide estimates in each one of the geographical strata, with an approximate maximum error of 3%, in absolute values with 95% confidence level. According to the School Census which was valid at the time of the research planning, all the 9th grade students enrolled in morning or afternoon shifts of the Elementary Schools was considered. In Brazil, students attend school in two shifts either from early morning to around midday, or from around midday to late afternoon. Further details of the sampling design can be found elsewhere [19]. Those who declined to take part in the research or those who did not report their age or sex on the questionnaire (*n* = 229; 0.22%) were excluded.

### Measures

The health-related behaviors analyzed in the study included diet, PA, and SB. The surveys were completed using an electronic questionnaire in smartphones. The questionnaire was broadly based on the Global School-Based Student Health Survey, and Youth Risk Behavior Surveillance System [20, 21] with a pilot test being conducted in order to make adjustments and determine adequacy.

Diet was assessed using seven questions. Adolescents reported how often (one to seven days) in the last week they ate: green salad or vegetables, fruits, deep-fried empanadas, candies, soda, fast foods and ultra-processed food. Dietary patterns were identified by performing exploratory factor analysis using the weekly average number of days of intake for each of the 7 identified food group variables. Kaiser-Meyer-Olkin (KMO) measures of sampling adequacy and Bartlett’s Test of Sphericity were used to address the suitability of the factor structure. Factor structure is often considered acceptable if KMO is greater than 0.6 and *p*-value of Bartlett’s Test for Sphericity is less than 0.50 [22]. Two factors were extracted based on the screen plot and the factor structure was suitable (KMO = 0.72 and *p* < 0.001 for Bartlett’s Test) (see Additional file 2). An item was considered a component of a factor if its factor loading is greater than 0.6. The factor score for each pattern was constructed by summing observed (days/week) intakes of the component food items and divided by the number of food items. The two food groups were: 1) deep-fried empanadas, candies, soda, ultra-processed and fast foods (‘unhealthy diet’); and 2) green salads or vegetables and fruits (‘healthy diet’). The Brazilian Dietary Guidelines recommends a daily consumption of non-processed foods as fruits and vegetables, and avoiding the consumption of ultra-processed food. The Brazilian dietary guideline does not recommend a specific amount for these dietary groups However, the combination of high consumption of healthy foods and low consumption of unhealthy is considered an adequate diet [23].

Students’ PA was assessed using the following question: *In the past 7 days*, *without considering physical education class, how many days did you practice some physical activity like sports, dance, gym exercises, combat sports or other activity?* The answers ranged from none to seven days in a week. SB was measured using the following question: *In a regular day, how much time do you spend watching television, playing videogames, talking with friends or other sitting activities?* The response options ranged from one to nine hour a day.

Sociodemographic variables included sex, age, type of school (public and private) and maternal level of education (non-educated, elementary school [low], high school [medium], and higher education [high]).

### Statistical analysis

Statistics were performed using Stata statistical software, version 14 (Stata Inc., College Station, TX, USA) except cluster analysis. SPSS for Windows (version 23 SPSS Inc.; Chicago, IL, USA) was used for the two-step cluster analysis. In all inferential statistical procedures, the methodology for complex analysis and weighting was used to incorporate strata, primary sampling units, and sample weight.

Cluster analysis were based on four variables: (1) Diet (two variables): the number of days a week eating an unhealthy or a healthy diet; (2) PA: the numbers of days of activity a week; and (3) SB: the number of hours a day watching television, playing videogames, talking with friends or other sitting activities. The log-likelihood was the distance metric. The number of clusters were based on the best combination of low Bayesian Information Criterion (BIC), high ratio of distance measures and high ratio of BIC changes as well as meaningful conceptual considerations (see Additional file 3). The quality of the cluster solution in the total data set was analyzed by silhouette coefficient indicating cohesion and separation (the silhouette ranges from − 1 to + 1; a high value indicates that the object is well matched to its own cluster and poorly matched to neighboring clusters). The relative importance of each variable in the model was also observed. The predictor importance ranges from 0 to 1 (values close to 1 means relatively more important). All four variables reached values equal to 1. In order to observe possible subgroups differences, the procedures were repeated in subsets of younger (11–14 years old) and older (15–19 years old) adolescents. The final number of clusters was the same across younger and older adolescents (see Additional files 4, 5). The health-related behaviors were normally distributed across the clusters, and there was an equal within-group variance across the groups. To examine and confirm cluster profiles differences between diet, PA and SB, Analysis of Variance (ANOVA) with Tukey multiple comparison tests was used. Eta-squared effect sizes were also reported.

Multinomial logistic regression (crude and adjusted) was used, with values expressed in odds ratio (OR) and their respective 95% confidence intervals (95% CI), to verify the association between sociodemographic variables and the identified clusters. The health-risk cluster was the reference category and represented the negative combination of the three behaviors. The significance level was defined as *p* < 0.01.

## Results

### Sample characteristics

Characteristics of the sample are shown in Table 1. The sample consisted of 52,782 girls and 49,290 boys with a mean age of 14.28 ± 1.03 (SD) years-old. Over 40% of the sample had mothers with low educational level or non-educated, and over 80% of the students attended public schools. Students ate an unhealthy diet 2.6 ± 1.5 days/week and ate a healthy diet 3.3 ± 2.1 days/week; reported spending 2.5 ± 2.5 days/week in PA, and 4 ± 2.7 h/day in SB.

**Table 1:** Participant characteristic. PeNSE Brazil, 2015 (*n* = 102,072)

Variables^*^		
Sex % (95% CI)		
Male	48.72	(48.08; 49.34)
Female	51.28	(50.65; 51.91)
Age mean ± sd (range)	14.28 ± 1.03 (11, 19)	
Maternal level of education % (95% CI)		
Non-educated	28.62	(27.65; 29.60)
Elementary School	18.48	(17.83; 19.16)
High School	33.32	(32.49; 34.15)
Higher Education	19.58	(18.32; 20.89)
Type of school % (95%CI)		
Public	85.53	(83.35; 87.46)
Private	14.47	(12.54; 16.64)

^*^Weighted percentages and means

95% CI 95% confidence interval. *sd* standard deviation

### Cluster profiles

**Fig. 1:**
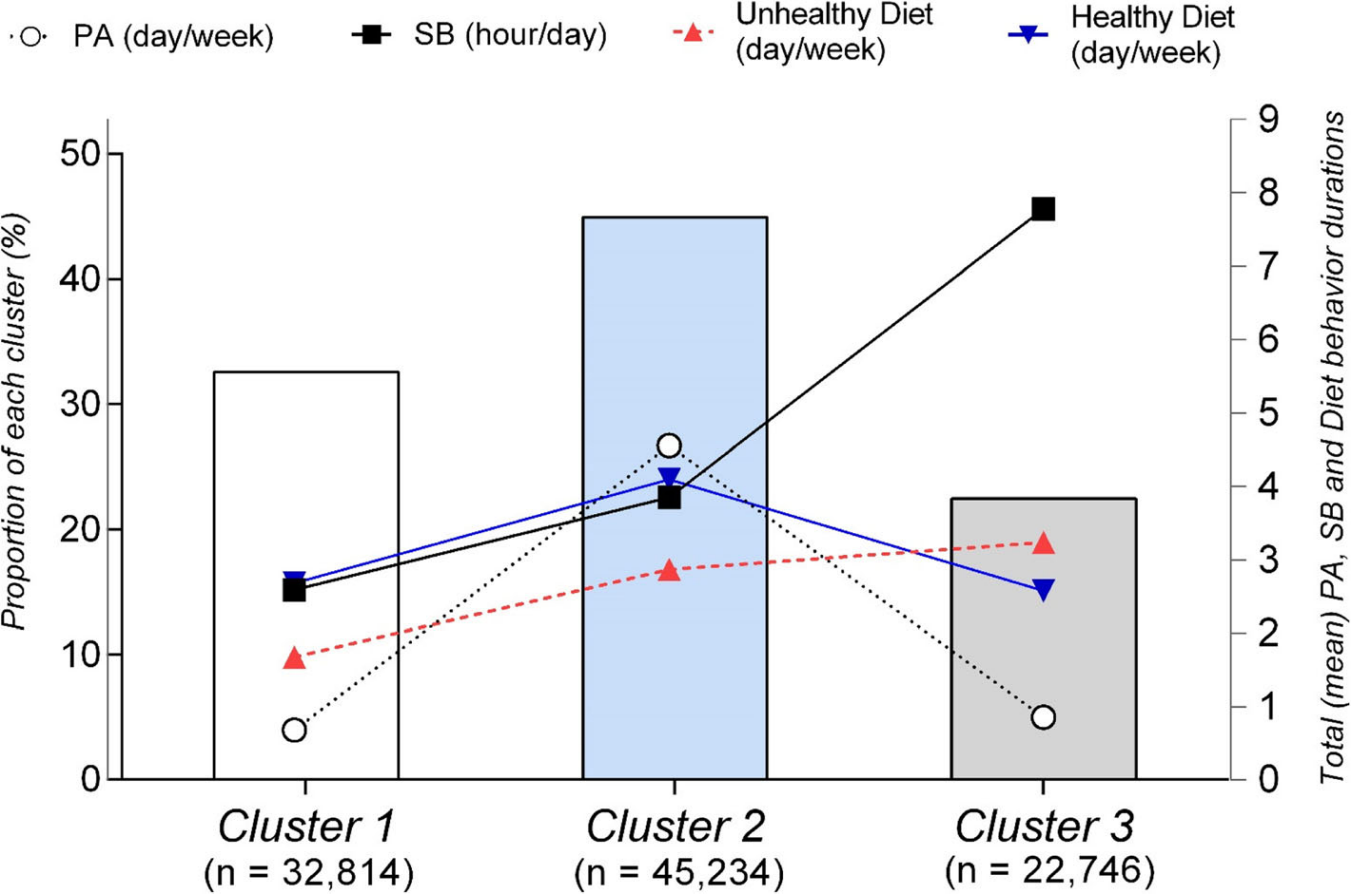
Physical activity (PA), sedentary behavior (SB), unhealthy diet and healthy diet in each of the three clusters. National School-Based Health Survey among ninth-grade students—PeNSE, 2015 (sample 1). Cluster 1: health-promoting SB and diet; cluster 2: health-promoting PA and diet; and cluster 3: health-risk

One hundred thousand seven hundred ninety four adolescent students were eligible for cluster analysis; 1278 were excluded for incomplete data. There were differences in sociodemographic variables between complete and incomplete data (see Additional file 6). Clusters were labelled according to the most accentuated behaviors and based on Azeredo et al. [24] work: cluster 1: health-promoting SB and diet; cluster 2: health-promoting PA and diet; and cluster 3: health-risk (Fig. 1). The silhouette coefficient was 0.40 in total sample, indicating a fair model.

Table 2 shows that PA, SB and, healthy and unhealthy diet differentiated among clusters in multivariate analysis (*p* < 0.001). PA and SB contributed most to the distinction between clusters (Eta-squared -largest effect size).

**Table 2:** Comparison of the three cluster solution for diet, PA and SB. PeNSE Brazil, 2015

	Cluster 1 Health-promoting SB and diet	Cluster 2 Health-promoting PA and diet	Cluster 3 Health-risk	F	*p* Value	Effect size
	*n* = 32,814	*n* = 45,234	*n* = 22,746			
	mean ± sd (range)	mean ± sd (range)	mean ± sd (range)			
Physical activity	0.68 ± 0.92 (0, 4)	4.56 ± 2.05 (0, 7)	0.86 ± 1.24 (0, 6)	72,102.5	*p* < 0.001	0.59
Sedentary behavior	2.59 ± 1.55 (1, 7)	3.85 ± 2.31 (1, 9)	7.78 ± 1.39 (3, 9)	52,596.5	*p* < 0.001	0.51
Unhealthy diet	1.68 ± 0.97 (0, 4.8)	2.87 ± 1.49 (0, 7)	3.24 ± 1.45 (0, 7)	11,387.9	*p* < 0.001	0.18
Healthy diet	2.68 ± 1.97 (0, 7)	4.10 ± 2.07 (0, 7)	2.58 ± 1.97 (0, 7)	6610.8	*p* < 0.001	0.16

*sd* standard deviation

Differences between clusters were observed by ANOVA test. All three clusters are significantly different at *p* < 0.001(Tukey post hoc)

Eta-squared effect sizes

The methodology for complex analysis and weighting was considered

The health-promoting SB and diet cluster (Cluster 1) comprised 32.6% (95% CI: 32.3–32.8) of the sample and was characterized by less than three hours a day in SB and less than two days/week of unhealthy diet. PA was reported less than 1 day/week and health dietary intake was 2.68 days/week. The health-promoting PA and diet cluster (Cluster 2) had the highest mean value for PA (more than five days/week) and SB was almost four hours/day. Participants reported just under 3 days/week eating an unhealthy diet and more than 4 days/week eating a healthy diet; this cluster comprised 44.9% (95% CI: 44.6–45.2) of the sample. The health-risk cluster (Cluster 3) represented almost a quarter of the sample (22.5%; 95% CI: 22.3–22.8) and included high levels of SB (almost eight hours/day) and a combination of unhealthy diet (more than three days/week) and less than 3 days/week of healthy diet; PA was less than one day/week.

### Sociodemographic characteristics associated with cluster profiles

Adolescents in the health-promoting SB and diet cluster were 14.42 ± 0.06 (SD) years old; most female (57.33%), from public schools (90.08%); over 50% of the maternal level of education were non-educated or elementary school. Adolescents in the health-promoting PA and diet cluster were 14.30 ± 0.47 years old, most males (58.65%) from public schools (83.29%) and the majority of the maternal level of education were high school. Those in the health-risk cluster were 14.25 ± 0.65 years old, most females (62.25%) from public schools (83.06%) and less than 20% of the adolescents had mothers with higher education (see Additional file 7).

Compared to boys, girls were 15% less likely to be in the health-promoting SB and diet cluster and 58% less likely to be in the health-promoting PA and diet cluster rather than the health-risk cluster (Table 3). For each additional year in age, participants were 7% more likely to be in the health-promoting SB and diet cluster. All levels of maternal education were inversely associated with health-promoting SB and diet clusters. Adolescents whose mothers had the highest level of maternal education were 37% less likely to be in the health-promoting SB and diet clusters. However, adolescents whose mothers had the highest level of maternal education were 21% more likely to be in the health-promoting PA and diet cluster rather that in the health-risk cluster. Students from private schools were 30% less likely to be in the health-promoting SB and diet cluster.

**Table 3:** Cluster’s profiles associated with sociodemographic characteristics. PeNSE Brazil, 2015

Variables	Health-promoting SB and diet				Health-promoting PA and diet			
	Crude		Adjusted		Crude		Adjusted	
	OR	95% CI	OR	95% CI	OR	95% CI	OR	95% CI
Sex								
Male	1		1		1		1	
Female	0.81**	(0.76;0.87)	0.85**	(0.78;0.92)	0.42**	(0.40;0.45)	0.43**	(0.40;0.46)
Age (mean ± sd)	1.17**	(1.14;1.21)	1.07**	(1.03;1.11)	1.05*	(1.02;1.08)	1.00	(0.96;1.04)
Maternal level of education								
Non-educated	1		1		1		1	
Elementary School	0.70**	(0.66;0.83)	0.76**	(0.68;0.85)	0.97	(0.88;1.08)	0.95	(0.85;1.05)
High School	0.60**	(0.54;0.67)	0.64**	(0.58;0.71)	0.96	(0.88;1.05)	0.95	(0.87;1.03)
Higher Education	0.53**	(0.47;0.60)	0.63**	(0.55;0.71)	1.21**	(1.09;1.35)	1.22**	(1.09;1.37)
Type of school								
Public	1		1		1		1	
Private	0.54**	(0.49;0.59)	0.70**	(0.63;0.79)	0.98	(0.89;1.05)	0.9	(1.09;1.37)

The health-risk cluster is the reference group in multinomial logistic regression

*OR* odds ratio, 95% CI confidence interval, *sd* standard deviation

Models adjusted for sex, age, maternal level of education and type of school

* Significant at *p* < 0.01, ** Significant at *p* < 0.001

## Discussion

This study examined the patterns of diet, PA and SB and its sociodemographic correlates among a national population-based sample of Brazilian adolescents. Three clusters were acknowledged. Our results recognized two as the mostly healthy clusters, one mainly characterized by low levels of SB and adequate diet (low consumption of unhealthy food), and other characterized as the highest levels of PA and adequate diet. The third cluster, comprised approximately one-quarter of the sample and was characterized as the unhealthiest group (low PA, high SB, and unhealthy diet). Identifying clusters of health behavior in youth is important for understanding the relationships between different lifestyle behaviors.

We found that behavioral risk factors are prevalent among Brazilian adolescents and it is important to mention that all clusters consisted of at least one unhealthy behavior. Therefore, we confirm the co-occurrence of positive and negative health-related behavior among Brazilian adolescents. The complexity of adolescents’ behavior was observed in Leech et al. [5] review, which the synergy between healthy and unhealthy behaviors were consistent among all studies reviewed; beyond adolescence characteristics where many behavioral adjustment appears, it was observed a high prevalence of clusters defined by high levels of SB. In this case, SB also appeared to be an issue for Brazilian adolescents.

The health-risk cluster reported the lowest frequency of PA (less than one day a week), spent almost eight hours a day in SB, and also reported eating unhealthy diet more than 3 days a week. Similar clusters of health-related behaviors have been reported in previous research from other countries [5, 13, 25]. Dumuid et al. [15] also verified a general profile of unhealthy clusters in low- and middle-income countries (LMIC), which might be attributable to the demographic transitions and the Westernization of these contexts. It is a worrisome picture, since exposure to clusters of unhealthy lifestyle habits over long periods of time may be associated with short- and long-term health risk factors which are the precursors of chronic disease.

Previous studies have shown a co-occurrence of higher SB and unhealthy diet [5, 9]. In our study we verified a cluster which those adolescents with lower SB, also had a lower consumption of unhealthy diet (health-promoting SB and diet cluster). Even with a good balance pattern between diet and SB, our cluster showed lower level of PA. Indeed, clusters composed by lower levels of SB and low intakes of unhealthy food, along with higher levels of PA have been found for adolescents from high income countries [5, 8], and not for those from LMIC, including Brazil [15]. However, as we observed, the HELENA-Cross Sectional Study, which was conducted with adolescents from 10 European cities, observed a cluster in which low sedentary levels and high diet quality appears with low PA levels [4].

The “health-promoting PA and diet cluster” confirmed systematic reviews of observational studies that reported a co-occurrence of PA and healthy diet markers [5, 9]. In the same way, Leech et al. [8] also observed in children (10–12 years-old) that the healthiest cluster was characterized by the lowest intakes of unhealthy food and the most time in moderate to vigorous PA. However, different from our results, they also identified the least time being sedentary. The literature remains inconsistent with some observational studies showing that clusters with high levels of PA are characterized by low levels of SB or screen time [26–28], whereas others reported a coexistence of high levels of both in the same cluster [5, 26]. Dumuid et al. [15] observed, among samples of children from twelve different countries, that the cluster of high screen time and high PA appears only for Brazilians, corroborating our results. These unique findings suggest that cluster patterns might be related to the culture of particular countries [8, 12].

Sociodemographic characteristics correlates were differentially associated with each of the clusters. We point an urgent need of health promotion engaging girls, who had a greater chance of being in the health-risk cluster. Several studies have shown that active clusters were predominantly composed of boys [5, 9, 26]. However, the fact that the Brazilian girls were more likely to also have unhealthy levels of diet and SB is not consistent with studies from other countries [5, 9]. A recent work with Brazilian adolescents (PeNSE 2012), using exploratory factor analysis to identify patterns of health behavior, also observed that a health compromising diet and SB cluster was associated with female gender [24]. These patterns of results may be a trend for Brazilians and the risk for those variables is established as it seems that boys and girls may be socialized in different ways and tend to have different encouragement to pursuit a healthy lifestyle [24, 29]. In this sense, a greater risk observed for girls may be the result of society’s expectations.

Increases in age were associated with being in the health-promoting SB and diet cluster. This was not consistent with findings from others studies that verified the healthiest clusters for diet, PA and SB to be associated with younger children and younger adolescents [5, 30, 31]. Higher levels of parental education were associated with clusters mainly characterized by high participation in PA [5], while low parental education with clusters mainly characterized by high SB. Usually socioeconomic status is positively associated with healthy patterns [9]. In the present study, this was confirmed for the health-promoting PA and diet cluster but not for the health-promoting SB and diet cluster.

The observed negative association between private schools and the health-promoting SB and diet cluster is consistent with recent studies with Brazilian adolescents [24, 29]. Private schools in Brazil are an indicative of individual socioeconomic advantage compared to public schools. It is observed that a socioeconomic advantage in LMIC can be an opportunity to access unhealthy behaviors and Brazilian adolescents from private schools can adopt both healthy and unhealthy behaviors. A recent study found that a great proportion of private schools (94.7%) have cafeterias among its facilities (compared to 35.1% in public schools). These cafeterias sell both healthy (soft drink, snacks, sweets) and unhealthy food (fruit and juice) [32]. Otherwise, students from public schools tend to have a healthier diet at school since they are submitted to the school dietary plans which the main goal is to promote a healthy and well-balance diet. These actions do not include private schools [33]. It seems that private schools are an indicative of social advantage were adolescents might have more opportunities to access environments that favor the adoption of a sedentary lifestyle [34]. The private schools might also encourage the students to be involved in screen-based activities aimed at educational, information technology and leisure purposes [35]. Indeed, the availability of computers and internet was greater in private schools when compared to public schools (79% vs 71 and 85% vs 72%, respectively) (data not shown).

This study has some limitations. Data are cross-sectional and the biases that accompany cross-sectional cluster analysis study are well known [8]. However, the sample comprised a large representative number of adolescent students from Brazil, a middle-income country, which is scarce in literature. However, it is important to note that only adolescents with completing data for the health-related outcomes were analyzed. Exclude participants might have particular characteristics that may bias the cluster formation. Analyses were based on self-reported measures which could result in participants’ answers being influenced by social desirability. The types of variables used in cluster analyses differ across studies making it difficult to compare with the literature. Also, in our approach SB consisted of different patterns of behavior as screen time and homework; however, these behaviors could be in different directions and the complexity of SB implies that we cannot assume an overall measure to indicate a healthy or unhealthy lifestyle. Regarding diet assessment and our complex sampling method, it is important to mention that exploratory factor analysis was conducted without taking into account the methodology for complex analysis. Two-step cluster analysis was also conducted without consideration of complex sampling scheme, which should be addressed as an important limitation. Also, a distinguishing clustering structure on the data set could be acknowledged by different clustering methods and the extrapolation of the results must be done carefully.

Among the possibilities of clustering analysis, our work shows some positive aspects, it allowed the analysis of a national and representative sample, observing which behaviors coexist among Brazilian adolescents. This study may provide important insights as analyses allowed the investigation of various association between the identified clusters and sociodemographic variables. That may contribute to a comprehensive understanding of which adolescent groups need more attention.

## Conclusions

Few studies have examined how health behaviors cluster among youth from LMIC. Three clusters were identified in a nationally representative sample of Brazilian adolescents: health-promoting SB and diet; health-promoting PA and diet, and health-risk cluster which was characterized by the lowest levels of PA, diet and SB, and comprised approximately one-quarter of adolescents in the sample. We observed that there were associations between clusters and sociodemographic groups, reinforcing that interventions may need to be tailored to specific adolescent groups, especially considering sociodemographic differences between boys and girls and socioeconomic groups.

## Additional files

Additional file 1: Frequency and proportion of adolescents according to age. PeNSE Brazil, 2015 (*n* = 102,072). (DOCX 13 kb)

Additional file 2: Factor loadings - exploratory factor analysis. Additional file shows dietary patterns. (DOCX 40 kb)

Additional file 3: Comparison of models by Bayesian Information Criterion and Ratio of Distance Measures in total sample. PeNSE Brazil, 2015 (*n* = 100,794). Additional file 3 shows the cluster solution (total sample) based on the best combination of low Bayesian Information Criterion (BIC), high ratio of distance measures and high ratio of BIC changes. (DOCX 15 kb)

Additional file 4: a: Comparison of models by Bayesian Information Criterion and Ratio of Distance Measures in younger adolescents. PeNSE Brazil, 2015 (*n* = 68,135). Additional file a shows the cluster solution (younger adolescents) based on the best combination of low Bayesian Information Criterion (BIC), high ratio of distance measures and high ratio of BIC changes. b: Comparison of the three cluster solution for diet, physical activity and sedentary behavior among younger adolescents. PeNSE Brazil, 2015 (*n* = 68,135). (DOCX 19 kb)

Additional file 5: a: Comparison of models by Bayesian Information Criterion and Ratio of Distance Measures in older adolescents. PeNSE Brazil, 2015 (*n* = 32,659). Additional file a shows the cluster solution (older adolescents) based on the best combination of low Bayesian Information Criterion (BIC), high ratio of distance measures and high ratio of BIC changes. b: Comparison of the three cluster solution for diet, physical activity and sedentary behavior among older adolescents. PeNSE Brazil, 2015 (*n* = 32,659). (DOCX 18 kb)

Additional file 6: Characteristic of excluded and included participants in cluster formation PeNSE Brazil, 2015. Additional file shows the differences in sociodemographic variables between subjects with complete and incomplete data. (DOCX 16 kb)

Additional file 7: Cluster’s profiles associated with sociodemographic characteristics. PeNSE Brazil, 2015. Additional file shows the distributions of the three clusters by each sociodemographic variable. (DOCX 68 kb)

## Abbreviations

95%CI: 95% confidence interval
ANOVA: analysis of variance
BIC: Bayesian information criterion
IBGE: Institute of geographic and statistics of Brazil
LMIC: low- and middle-income countries
MEC: Ministry of education
OR: Odds ratio
PA: physical activity
PeNSE: national school-based health
SB: sedentary behavior
WHO: world health organization
