# Supplementary material for: Clustering of diet, physical activity and sedentary behavior among Brazilian adolescents in the national school - based health survey (PeNSE 2015)
Source: BMC Public Health. 2018 Nov 21;18:1283. doi: 10.1186/s12889-018-6203-1 (PMC6249930; doi:10.1186/s12889-018-6203-1)
Supplement: Supplementary file 1 — Frequency and proportion of adolescents according to age. PeNSE Brazil, 2015 (n = 102,072). (DOCX 13 kb) [file 12889_2018_6203_MOESM1_ESM.docx]

| Additional file 1. Frequency and proportion of adolescents according to age. PeNSE Brazil, 2015 (n=102,072). | | |
| --- | --- | --- |
| Age (years) | n | % |
| 11 | 28 | 0.05 |
| 12 | 510 | 0.36 |
| 13 | 16,722 | 17.84 |
| 14 | 51,611 | 51.01 |
| 15 | 20,864 | 19.78 |
| 16 | 7,873 | 7.18 |
| 17 | 3,040 | 2.54 |
| 18 | 852 | 0.75 |
| 19 | 572 | 0.49 |
