# Supplementary material for: Clustering of diet, physical activity and sedentary behavior among Brazilian adolescents in the national school - based health survey (PeNSE 2015)
Source: BMC Public Health. 2018 Nov 21;18:1283. doi: 10.1186/s12889-018-6203-1 (PMC6249930; doi:10.1186/s12889-018-6203-1)
Supplement: Supplementary file 2 — Factor loadings - exploratory factor analysis. Additional file shows dietary patterns. (DOCX 40 kb) [file 12889_2018_6203_MOESM2_ESM.docx]

| Additional file 2. Factor loadings - exploratory factor analysis. | | |
| --- | --- | --- |
|  | Component | |
| Predictor | 1 | 2 |
| Deep-fried empanadas | 0.71 | -0.08 |
| Green salad or vegetables | 0.10 | 0.83 |
| Candies | 0.64 | -0.09 |
| Fruits | 0.22 | 0.80 |
| Soda | 0.67 | -0.16 |
| Ultra-processed food | 0.67 | -0.09 |
| Fast food | 0.63 | 0.04 |
| Extraction method: Principal Component Analysis. Two components were extracted. | | |
|  |  |  |
| An item was considered a component of a factor if its factor loading is greater than 0.6. | | |
