# Supplementary material for: Clustering of diet, physical activity and sedentary behavior among Brazilian adolescents in the national school - based health survey (PeNSE 2015)
Source: BMC Public Health. 2018 Nov 21;18:1283. doi: 10.1186/s12889-018-6203-1 (PMC6249930; doi:10.1186/s12889-018-6203-1)
Supplement: Supplementary file 3 — Comparison of models by Bayesian Information Criterion and Ratio of Distance Measures in total sample. PeNSE Brazil, 2015 (n = 100,794). Additional file 3 shows the cluster solution (total sample) based on the best combination of low Bayesian Information Criterion (BIC), high ratio of distance measures and high ratio of BIC changes. (DOCX 15 kb) [file 12889_2018_6203_MOESM3_ESM.docx]

| Additional file 3. Comparison of models by Bayesian Information Criterion and Ratio of Distance Measures in total sample. PeNSE Brazil, 2015 (n=100,794). | | | | |
| --- | --- | --- | --- | --- |
| Number of Clusters | BIC | BIC Change* | Ratio of BIC Changes** | Ratio of Distance Measures*** |
| 1 | 279550.474 |  |  |  |
| 2 | 230359.27 | -49191.204 | 1 | 1.665 |
| **3** | **200845.719** | **-29513.551** | **0.6** | **1.755** |
| 4 | 184068.613 | -16777.106 | 0.341 | 1.343 |
| 5 | 171599.279 | -12469.334 | 0.253 | 1.207 |
| 6 | 161280.571 | -10318.709 | 0.21 | 1.041 |
| 7 | 151369.164 | -9911.407 | 0.201 | 1.087 |
| 8 | 142256.113 | -9113.051 | 0.185 | 1.455 |
| 9 | 136023.738 | -6232.375 | 0.127 | 1.294 |
| 10 | 131229.524 | -4794.214 | 0.097 | 1.175 |
| 11 | 127164.253 | -4065.271 | 0.083 | 1.031 |
| 12 | 123222.405 | -3941.848 | 0.08 | 1.068 |
| 13 | 119536.421 | -3685.984 | 0.075 | 1.059 |
| 14 | 116061.695 | -3474.726 | 0.071 | 1.007 |
| 15 | 112610.589 | -3451.106 | 0.07 | 1.134 |
| BIC: Bayesian Information Criterion. | |  |  |  |
| * The changes are from the previous number of clusters in the table. | | | |  |
| ** The ratios of changes are relative to the change for the two cluster solution. | | | |  |
| *** The ratios of distance measures (log-likelihood) are based on the current number of clusters against the previous number of clusters. | | | | |
