# Supplementary material for: Clustering of diet, physical activity and sedentary behavior among Brazilian adolescents in the national school - based health survey (PeNSE 2015)
Source: BMC Public Health. 2018 Nov 21;18:1283. doi: 10.1186/s12889-018-6203-1 (PMC6249930; doi:10.1186/s12889-018-6203-1)
Supplement: Supplementary file 4 — a: Comparison of models by Bayesian Information Criterion and Ratio of Distance Measures in younger adolescents. PeNSE Brazil, 2015 (n = 68,135). Additional file a shows the cluster solution (younger adolescents) based on the best combination of low Bayesian Information Criterion (BIC), high ratio of distance measures and high ratio of BIC changes. b: Comparison of the three cluster solution for diet, physical activity and sedentary behavior among younger adolescents. PeNSE Brazil, 2015 (n = 68,135). (DOCX 19 kb) [file 12889_2018_6203_MOESM4_ESM.docx]

| Additional file 4a. Comparison of models by Bayesian Information Criterion and Ratio of Distance Measures in younger adolescents. PeNSE Brazil, 2015 (n=68,135). | | | | |
| --- | --- | --- | --- | --- |
| Number of Clusters | BIC | BIC Change* | Ratio of BIC Changes** | Ratio of Distance Measures*** |
| 1 | 188997.367 | - | - | - |
| 2 | 155036.628 | -33960.739 | 1 | 1.781 |
| **3** | **136004.951** | **-19031.677** | **0.56** | **1.836** |
| 4 | 125681.819 | -10323.132 | 0.304 | 1.084 |
| 5 | 116163.57 | -9518.248 | 0.28 | 1.219 |
| 6 | 108369.658 | -7793.912 | 0.229 | 1.284 |
| 7 | 102317.667 | -6051.992 | 0.178 | 1.183 |
| 8 | 97214.8 | -5102.867 | 0.15 | 1.392 |
| 9 | 93573.361 | -3641.439 | 0.107 | 1.071 |
| 10 | 90179.603 | -3393.757 | 0.1 | 1.03 |
| 11 | 86888.741 | -3290.862 | 0.097 | 1.025 |
| 12 | 83680.091 | -3208.651 | 0.094 | 1.178 |
| 13 | 80969.838 | -2710.253 | 0.08 | 1.127 |
| 14 | 78574.665 | -2395.174 | 0.071 | 1.069 |
| 15 | 76339.133 | -2235.532 | 0.066 | 1.251 |
| \| BIC: Bayesian Information Criterion. \|  \|  \|  \| \| --- \| --- \| --- \| --- \| | | | | |
| * The changes are from the previous number of clusters in the table. | | | | |
| ** The ratios of changes are relative to the change for the two cluster solution. | | | | |
| *** The ratios of distance (log-likelihood) measures are based on the current number of clusters against the previous number of clusters. | | | | |

| Additional file 4b. Comparison of the three cluster solution for diet, PA and SB among younger adolescents. PeNSE Brazil, 2015 (n=68,135). | | | | | | | | | | |
| --- | --- | --- | --- | --- | --- | --- | --- | --- | --- | --- |
|  |  | **Cluster 1** | |  | **Cluster 2** |  | **Cluster 3** |  |  |  |
|  |  | | Health-promoting |  | Health-promoting |  | Health-risk |  |  |  |
|  |  | | SB and diet |  | PA and diet |  |  | F | *p* | Effect |
|  |  | | n=30,103 |  | n=22,323 |  | n=15,709 |  | Value | size |
|  | | | mean ± sd (range) |  | mean ± sd (range) |  | mean ± sd (range) |  |  |  |
| Physical activity | | | 1.07±1.16 (0, 4) |  | 5.47±1.39 (1, 7) |  | 0.86±1.18 (0, 5) | 96392.1 | p<0.001 | 0.74 |
| Sedentary behavior | | | 2.89±1.54 (1, 7) |  | 4.17±2.45 (1, 9) |  | 7.85±1.25 (4, 9) | 37863.8 | p<0.001 | 0.53 |
| Unhealthy diet | | | 2.14±1.24 (0, 7) |  | 2.73±1.48 (0, 7) |  | 3.19±1.44 (0, 7) | 3252.5 | p<0.001 | 0.09 |
| Healthy diet | | | 3.00±1.99 (0, 7) |  | 4.30±2.13 (0, 7) |  | 2.78±2.03 (0, 7) | 3480.5 | p<0.001 | 0.09 |
| sd = standard deviation. | | | |  |  |  |  |  |  |  |
| Differences between clusters were observed by ANOVA test. All three factors were significantly different at p<0.001(Tukey Post Hoc). | | | | | | | | | | |
| Eta-squared effect sizes. | | | |  |  |  |  |  |  |  |
