# Supplementary material for: Clustering of diet, physical activity and sedentary behavior among Brazilian adolescents in the national school - based health survey (PeNSE 2015)
Source: BMC Public Health. 2018 Nov 21;18:1283. doi: 10.1186/s12889-018-6203-1 (PMC6249930; doi:10.1186/s12889-018-6203-1)
Supplement: Supplementary file 5 — a: Comparison of models by Bayesian Information Criterion and Ratio of Distance Measures in older adolescents. PeNSE Brazil, 2015 (n = 32,659). Additional file a shows the cluster solution (older adolescents) based on the best combination of low Bayesian Information Criterion (BIC), high ratio of distance measures and high ratio of BIC changes. b: Comparison of the three cluster solution for diet, physical activity and sedentary behavior among older adolescents. PeNSE Brazil, 2015 (n = 32,659). (DOCX 18 kb) [file 12889_2018_6203_MOESM5_ESM.docx]

| Additional file 5a. Comparison of models by Bayesian Information Criterion and Ratio of Distance Measures in older adolescents. PeNSE Brazil, 2015 (n=32,659). | | | | |
| --- | --- | --- | --- | --- |
| Number of Clusters | BIC | BIC Change* | Ratio of BIC Changes** | Ratio of Distance Measures*** |
| 1 | 90631.126 | - | - | - |
| 2 | 77479.44 | -13151.686 | 1 | 1.422 |
| **3** | **68255.931** | **-9223.509** | **0.701** | **1.555** |
| 4 | 62355.7 | -5900.232 | 0.449 | 1.176 |
| 5 | 57352.023 | -5003.677 | 0.38 | 1.308 |
| 6 | 53547.576 | -3804.447 | 0.289 | 1.147 |
| 7 | 50241.167 | -3306.409 | 0.251 | 1.197 |
| 8 | 47493.149 | -2748.019 | 0.209 | 1.122 |
| 9 | 45052.074 | -2441.075 | 0.186 | 1.221 |
| 10 | 43067.91 | -1984.164 | 0.151 | 1.167 |
| 11 | 41380.276 | -1687.634 | 0.128 | 1.035 |
| 12 | 39752.837 | -1627.439 | 0.124 | 1.077 |
| 13 | 38247.055 | -1505.782 | 0.114 | 1.081 |
| 14 | 36860.225 | -1386.83 | 0.105 | 1.35 |
| 15 | 35854.369 | -1005.856 | 0.076 | 1.027 |
| BIC: Bayesian Information Criterion. | | | | |
| * The changes are from the previous number of clusters in the table. | | | | |
| ** The ratios of changes are relative to the change for the two cluster solution. | | | | |
| *** The ratios of distance measures (log-likelihood) are based on the current number of clusters against the previous number of clusters. | | | | |

| Additional file 5b. Comparison of the three cluster solution for diet, PA and SB among older adolescents. PeNSE Brazil, 2015 (n=32,659). | | | | | | | | | | | | | | | | |  |
| --- | --- | --- | --- | --- | --- | --- | --- | --- | --- | --- | --- | --- | --- | --- | --- | --- | --- |
|  |  | **Cluster 1** |  | | **Cluster 2** | |  | **Cluster 3** | | | |  | |  | |  | |
|  |  | Health-promoting |  | | Health-promoting | |  | Health-risk | | | |  | |  | |  | |
|  |  | SB and diet |  | | PA and diet | |  |  | | | | F | | *p* | | Effect | |
|  |  | n=12,975 |  | | n=11,677 | |  | n=8,007 | | | |  | | Value | | size | |
|  | | mean ± SD (range) |  | | mean ± SD (range) | |  | mean ± SD (range) | | | |  | |  | |  | |
| Physical activity | | 0.82±1.03 (0, 4) |  | | 5.39±1.53 (0, 7) | |  | 0.83±1.15 (0, 5) | | | | 49297.4 | | p<0.001 | | 0.75 | |
| Sedentary behavior | | 2.13±1.27 (1, 6) |  | | 3.94±2.53 (1, 9) | |  | 7.43±1.59 (3, 8) | | | | 19593.7 | | p<0.001 | | 0.54 | |
| Unhealthy diet | | 1.89±1.25 (0, 7) |  | | 2.85±1.61 (0, 7) | |  | 3.16±1.55 (0, 7) | | | | 2276.0 | | p<0.001 | | 0.12 | |
| Healthy diet | | 2.62±2.04 (0, 7) |  | | 3.87±2.13 (0, 7) | |  | 2.86±2.07 (0, 7) | | | | 1191.4 | | p<0.001 | | 0.07 | |
| sd = standard deviation. | | | |  |  | | | |  |  |  | |  | |  | |  |
| Differences between clusters were observed by ANOVA test. All three factors are significantly different at p<0.001(Tukey post hoc). | | | | | | | | | | | | | | | | |  |
| Eta-squared effect sizes. | | | |  | |  | | |  |  |  | |  | |  | |  |
