# Supplementary material for: Clustering of diet, physical activity and sedentary behavior among Brazilian adolescents in the national school - based health survey (PeNSE 2015)
Source: BMC Public Health. 2018 Nov 21;18:1283. doi: 10.1186/s12889-018-6203-1 (PMC6249930; doi:10.1186/s12889-018-6203-1)
Supplement: Supplementary file 6 — Characteristic of excluded and included participants in cluster formation PeNSE Brazil, 2015. Additional file shows the differences in sociodemographic variables between subjects with complete and incomplete data. (DOCX 16 kb) [file 12889_2018_6203_MOESM6_ESM.docx]

| Additional file 6. Characteristic of excluded and included participants in cluster formation PeNSE Brazil, 2015. | | | | | | |  |
| --- | --- | --- | --- | --- | --- | --- | --- |
| Variables | Excluded (n=1,278) | |  | Included (n=100,794) | |  |  |
|  | %* | 95%CI |  | %* | 95%CI | *p-*value |  |
| Sex |  |  |  |  |  |  |  |
| Male | 57.57 | (50.58; 64.28) |  | 48.61 | (47.98; 49.25) |  |  |
| Female | 42.42 | (35.71; 49.41) |  | 51.38 | (50.74; 52.01) | 0.009^a^ |  |
| Age** (mean ± sd) | 14.56 ± 1.23 | |  | 14.28 ± 1.03 | | <0.001^b^ |  |
| Maternal level of education |  |  |  |  |  |  |  |
| Non-educated | 36.32 | (29.98; 43.18) |  | 28.54 | (27.57; 29.53) |  |  |
| Elementary School | 21.06 | (16.35; 26.70) |  | 18.46 | (17.80; 19.13) |  |  |
| High School | 30.36 | (24.64; 36.76) |  | 33.34 | (32.51; 34.18) |  |  |
| Higher Education | 12.24 | (8.42; 17.46) |  | 19.64 | (18.38; 20.97) | 0.009^a^ |  |
| Type of school |  |  |  |  |  |  |  |
| Public | 93.30 | (90.42; 95.36) |  | 85.39 | (93.38; 96.47) |  |  |
| Private | 6.69 | (4.63; 9.57) |  | 14.60 | (12.65; 16.79) | <0.001^a^ |  |
| * Weighted percentages and means; ** age-range: from 11 to 19 years. | | | | | | |  |
| 95%CI = 95% confidence interval. sd = standard deviation.  ^a^ Rao-Scott chi-square test; ^b^ two-sample t-test.  The methodology for complex analysis and weighting was considered. | | | | | | |  |
