# Supplementary material for: Clustering of diet, physical activity and sedentary behavior among Brazilian adolescents in the national school - based health survey (PeNSE 2015)
Source: BMC Public Health. 2018 Nov 21;18:1283. doi: 10.1186/s12889-018-6203-1 (PMC6249930; doi:10.1186/s12889-018-6203-1)
Supplement: Supplementary file 7 — Cluster’s profiles associated with sociodemographic characteristics. PeNSE Brazil, 2015. Additional file shows the distributions of the three clusters by each sociodemographic variable. (DOCX 68 kb) [file 12889_2018_6203_MOESM7_ESM.docx]

| Additional file 7. Cluster's profiles associated with sociodemographic characteristics. PeNSE Brazil, 2015. | | | | |
| --- | --- | --- | --- | --- |
| Variables | Health-promoting SB and diet | Health-promoting PA and diet | Health-risk |  |
|  | %* (95%CI) | % *(95%CI) | %* (95%CI) | *p*-value |
| Sex |  |  |  |  |
| Male | 42.67 (41.71;43.64) | 58.65 (57.72;59.58) | 37.75 (36.50;39.02) |  |
| Female | 57.33 (56.36;58.29) | 41.35 (40.42;42.28) | 62.25 (60.98;63.50) | <0.001^a^ |
| Age** (mean ± sd) | 14.42 ± 0.06 | 14.30 ± 0.47 | 14.25 ± 0.65 | <0.001^b^ |
| Maternal level of education |  |  |  |  |
| Non-educated | 36.02 (34.65;37.41) | 25.30 (24.17;26.45) | 25.95 (24.56;27.38) |  |
| Elementary School | 19.29 (18.30;20.31) | 17.84 (17.00;18.69) | 18.70 (17.54;19.91) |  |
| High School | 30.27 (29.02;31.35) | 33.86 (32.79;34.95) | 36.00 (34.54;37.48) |  |
| Higher Education | 14.42 (13.17;15.75) | 23.00 (21.42;24.65) | 19.35 (17.87;20.91) | <0.001^a^ |
| Type of school |  |  |  |  |
| Public | 90.08 (88.49;91.47) | 83.29 (80.71;85.58) | 83.07 (80.71;85.45) |  |
| Private | 9.92 (8.85;11.50) | 16.71 (14.41;19.28) | 16.93 (14.54;19.61) | <0.001^a^ |
| * Weighted percentages and means; ** age-range: from 11 to 19 years.  OR = odds ratio; 95%CI = confidence interval, sd = standard deviation.  a Rao-Scott chi-square test; b one-way analysis of variance.  The methodology for complex analysis and weighting was considered. | | | |  |
